# Upregulation of CD146 in Pediatric B-Cell Acute Lymphocytic Leukemia and Its Implications on Treatment Outcomes

**DOI:** 10.1155/2020/9736159

**Published:** 2020-02-08

**Authors:** Asmaa M. Zahran, Omnia El-Badawy, Khalid I. Elsayh, Wael M. Y. Mohamed, Khalid F. Riad, Mona H. Abdel-Rahim, Amal Rayan

**Affiliations:** ^1^Clinical Pathology Department, South Egypt Cancer Institute, Assiut University, Assiut, Egypt; ^2^Medical Microbiology & Immunology Department, Faculty of Medicine, Assiut University, Assiut, Egypt; ^3^Pediatric Department, Faculty of Medicine, Assiut University, Assiut, Egypt; ^4^Oncology Department, Faculty of Medicine, Port Said University, Port Said, Egypt; ^5^Pediatric Oncology Department, South Egypt Cancer Institute, Assiut University, Assiut, Egypt; ^6^Clinical Oncology Department, Assiut University Hospital, Assiut University, Assiut, Egypt

## Abstract

*Background and Aim*. We studied through flow cytometry the expression of CD146 on different T cells, and B-cell ALL blasts trying to correlate its expression with different prognostic factors of B-cell ALL and treatment outcomes. *Patients and Methods*. All pediatric patients with B-cell ALL were subjected to bone marrow examination and cytochemistry, flow cytometric immunophenotyping using monoclonal antibodies utilized for diagnosis of B-ALL including CD34, CD19, CD10, CD22, and intracellular IgM. The diagnosis was based on standard morphologic, cytochemical, and immunophenotypic followed by flow cytometric detection of CD146 expression on blast cells, CD4^+^, and CD8^+^ T cells.

**Results:**

Significant accumulations of CD146^+^CD4^+^ cells, CD146^+^CD8^+^ cells, CD4^+^, CD8^+^, and lymphocytes in patients were compared to controls, the mean percentages of CD146^+^CD4^+^ cells, CD146^+^CD8^+^ cells, and CD146^+^ blasts were significantly higher in patients than controls, and in addition, these cells were associated with poor overall survival and disease-free survival. The median OS for patients with complete response was 22 ± 1.633 (95%CI = 18.799‐25.201), while for those without complete response, it was 13 ± 3.928 (95%CI = 5.301‐25.699), with log‐rank = 5.71, *P* = 0.017.

**Conclusion:**

CD146 was expressed significantly in children's B-ALL and associated with poor prognostic features including poor response and treatment outcomes and could be a possible poor prognostic factor in pediatric B-cell ALL.

## 1. Introduction

Acute lymphoblastic leukemia (ALL) is by far the most common pediatric malignancy, representing one quarter of all children cancers. Annually, there are 3.7-4.9 cases of ALL per 100,000 children aged 0-14 years in USA [1].

The exact risk factors for developing ALL have yet defined; most studies revealed mixed results with both maternal and infant causes which have been accused including maternal age, birth weight and order, smoking, and pesticide exposure.

With improvements in diagnostic and treatment modalities, overall cure rates for pediatric ALL have reached 90% [2]; the use of risk-adapted treatment protocols has improved cure rates while minimizing the toxicity of therapy.

CD146, also known as Mel-CAM, MUC18, MCAM, S-Endo-1, and P1H12 antigen [3], is a transmembrane glycoprotein belonging to the immunoglobulin family and functions as a Ca^2+^-independent adhesion molecule [4]. It is expressed in normal tissues including smooth muscles, vascular endothelium, and others to exert cation-independent adhesion through interactions with an unidentified ligand on the surface of various cells [5]; further studies revealed that CD146 has multifunctional activities both in physiological and pathological conditions including immunity, angiogenesis, and development.

A growing number of studies suggested that CD146 overexpression was significantly correlated with progression, angiogenesis, and metastasis of different malignant tumors like esophageal cancer, melanoma, gallbladder adenocarcinoma, ovarian carcinoma, and prostate cancer [6–12].

Further studies proved its role in many solid tumors including breast cancer [13], lung cancer [14], colorectal cancer [15], and hepatocellular carcinoma [16].

In a recent meta-analysis [17], high CD146 expression in solid tumors was associated with poor survival and might be considered as a useful prognostic biomarker and promising therapeutic target for different solid tumors.

Paucity is known about the role of CD146 in hematopoietic cells, although CD146 expression could identify a unique subset of CD3^+^CD4^+^ T-lymphocytes that may play an important role in the pathogenesis of various musculoskeletal diseases; however, its expression on activated T-cell populations as well as on a subset of murine NK cells was also reported [18, 19].

Though the biologic role of CD146 in hematologic malignancies remains to be defined, one study showed a very low number of CD146-positive AML blasts (3.3% of AML) when compared to CD146-positive B-ALL. Interestingly, all CD146-positive AML cases were classified as secondary AML not otherwise specified. Conversely, 66% of T-ALL and 36.8% of the total B-ALL cases, comprising cases bearing the t(9;22)(q34;q11)/BCR/ABL translocation, expressed CD146 on their blasts [20].

The expression of CD146 has been shown to be higher among adult B-cell ALL compared with pediatric B-cell ALL correlating with CD117, and CD64-positive cells in the former, while correlating with CD71- and CD56-positive cells in the latter [21].

Hence, the improvement of immunotyping of these tumors is important for accurate diagnostic workup of ALL; so we study through flow cytometry the expression of CD146 on different T cells, and B-cell ALL blasts trying to correlate its expression with different prognostic factors of B-cell ALL and treatment outcomes.

## 2. Patients and Methods

This study was a prospective case-controlled study that included 31 patients with de novo ALL presented to the South Egypt Cancer Institute (SECI), Assiut University. Twenty-eight age- and sex-matched healthy controls were also included in the study. The study was approved by the Institutional Review Board of the SECI, Assiut University. An informed written consent was taken from of all cases and controls.

All patients and controls were subjected to as follows:

- Thorough history taking and clinical examination, with careful assessment of clinical signs relevant to leukemia as fever, bone pain, hepatomegaly, splenomegaly, and lymphadenopathy
- Complete blood pictures by Ruby Cell Dyn (American, serial number: 36026BG) and Cell Dyn 1700 (American, serial number: 513554)
- Flow cytometric detection of the CD146 expression on peripheral blood T cells

Only patients were subjected to as follows:

- Bone marrow examination and cytochemistry study
- Flow cytometric immunophenotyping using monoclonal antibodies that were used for diagnosis of ALL including CD34, CD19, CD10, CD22, and intracellular IgM. All monoclonal antibodies were purchased from Becton Dickinson (BD) Biosciences, CA, USA. The diagnosis was based on standard morphologic, cytochemical, and immunophenotypic data of the patients
- Flow cytometric detection of CD146 expression on blast cells

### 2.1. Flow Cytometric Detection of the CD146 Expression on Peripheral Blood T Cells

**Figure 1:**
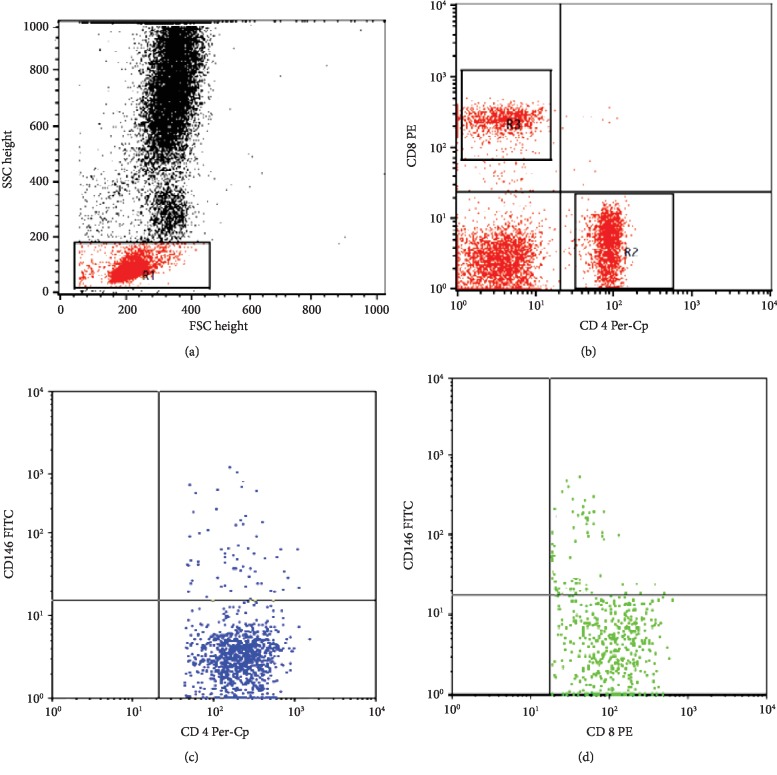
Flow cytometric detection of the CD146 expression on peripheral blood T cells. (a) Forward and side scatter histogram was used to define the lymphocyte population (R1). (b) The expressions of CD4 and CD8 on the lymphocyte population were detected, and then, CD4^+^ cells and CD8^+^ cells were gated for further analysis of CD146. (c) The expression of CD146 on CD4^+^ cells. (d) The expression of CD146 on CD8^+^ cells.

Peripheral blood mononuclear cells (PBMCs) were isolated from peripheral blood by Ficoll density gradient centrifugation (Biochrom GmbH, Germany). The cells were washed, and 2 × 10^6^ cells were stained with fluoroisothiocyanate- (FITC-) conjugated-CD146, phycoerythrin- (PE-) conjugated-CD8, and peridinium-chlorophyll-protein- (Per-CP-) conjugated-CD4 (all from BD Biosciences, CA, USA) for 20 min at 4°C in the dark. After one wash, the cells were suspended in phosphate-buffered saline (PBS) and analyzed by FACSCalibur flow cytometry with CellQuest software (BD Biosciences, USA). An isotype-matched negative control was used for each sample. Forward and side scatter histogram was used to define the lymphocyte population. Then, CD4^+^ cells and CD8^+^ cells were gated. Then, the expression of CD146 on CD4^+^ cells (CD146/CD4^+^) cells and on CD8^+^ cells (CD146/CD8^+^) was detected. The results of CD146 were expressed as percentage of CD4^+^ and CD8^+^cells as shown in Figure 1.

### 2.2. Flow Cytometric Detection of the CD146 Expression on Blast Cells

**Figure 2:**
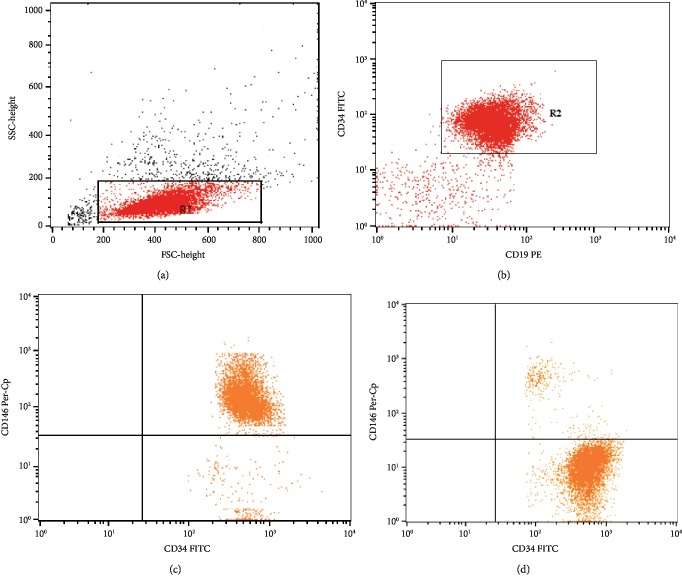
Flow cytometric detection of the CD146 expression on lymphoblasts. (a) Forward and side scatter histogram was used to define the blast cells (R1). (b) The expressions of CD19 and CD34 were assessed in blast cells to confirm lymphoblast gating which were then gated for further expression of CD146. (c) A patient with high expression of CD146 on CD19^+^CD34^+^ lymphoblasts. (d) A patient with low expression of CD146 on CD19^+^CD34^+^ lymphoblasts.

Fifty *μ*l of bone marrow sample were incubated with 5 *μ*l of FITC-anti-CD34, PE-anti-CD19, and Per-CP-anti-CD146 (all from BD Biosciences, CA, USA) for 20 min at 4°C in the dark. Following incubation, red blood cell lysis and washing with PBS were done. After washing, the cells were resuspended in PBS and analyzed by FACSCalibur flow cytometry with CellQuest software (BD Biosciences, USA). An isotype-matched negative control was used for each sample. Forward and side scatter histogram was used to define the blast cell population. The blast cells were gated for further analysis of the expression of CD34 and CD19. Then, the expression of CD146 was detected in CD34^+^CD19^+^ lymphoblasts. CD146 expression was defined by the percentage of lymphoblasts (Figure 2).

### 2.3. Statistics

Statistical package for social sciences (SPSS) version 20 was used for data analysis. All quantitative data were expressed as mean ± standard deviation (SD). Differences in a mean between the different groups of subjects were calculated using the independent sample *t*-test. Pearson correlation was used to detect the relation between different quantitative variables, the Kaplan-Meier test was used to graph the survival curves of different response groups, and the log-rank test was used to find a difference; Cox regression for survival analysis was used to investigate the effect of different variables on the survival. *P* value < 0.05 was considered significant. Overall survival (OS) was defined as the time interval between diagnosis and death of any cause, while disease-free survival (DFS) was set as time elapsed between diagnosis and relapse.

## 3. Results

This study involved pediatric patients with B-cell ALL having a median age of 6.5 years; as known for pediatric tumors, male children are exceedingly affected than female ones, so we had a male to female ratio of 1.4 : 1. Most patients were presented with constitutional symptoms including fever in (16/31) patients, whereby their caregivers pursued medical care, hepatomegaly, LN enlargement, and splenomegaly which were presented in 32.3%, 25.8%, and 22.6% of study patients (Table 1).

28 control children of comparable age and sex were taken for comparison of their immune cells with peers of B-cell ALL where significant accumulations of CD146^+^CD4^+^ cells, CD146^+^CD8^+^ cells, CD4^+^, CD8^+^, and lymphocytes were evidenced as illustrated in Table 2.

Upon correlating different immune cells carrying CD146 antigen with each other and with other prognostic factors, we detected positive significant correlations between CD146^+^CD4^+^ cells with CD146^+^CD8^+^ cells (*r* = +0.999, *P* < 0.001), BM blasts (*r* = +0.490, *P* = 0.005), CD146^+^ blasts (*r* = +0.910, *P* < 0.001), and CD34^+^ cells (*r* = +0.422, *P* = 0.017). Similarly, CD146^+^CD8^+^ cells correlated significantly with BM blasts (*r* = +0.494, *P* = 0.001), CD34^+^ cells (*r* = +0.420, *P* = 0.018), and CD146^+^ blasts (*r* = +0.932, *P* < 0.006). In addition, CD146 blasts exhibited significantly positive correlations with BM blasts (*r* = +0.528, *P* = 0.002), and CD34^+^ cells (*r* = +0.405, *P* = 0.024) (Table 3).

### 3.1. Relation of CD146^+^ Cells to the Response of Treatment

Significant differences in the mean percentages of CD146^+^CD4^+^ cells, CD146^+^CD8^+^ cells, and CD146^+^ blasts between those attained complete response to induction chemotherapy and those with less than complete response implicating that CD146 is considered a poor prognostic factor for primary response to induction therapy in B-cell ALL (Table 4).

### 3.2. Correlations between CD146^+^CD4^+^ Cells, CD146^+^CD8^+^ Cells, CD146^+^ Blasts, and Overall Survival and Disease-Free Survival

**Figure 3:**
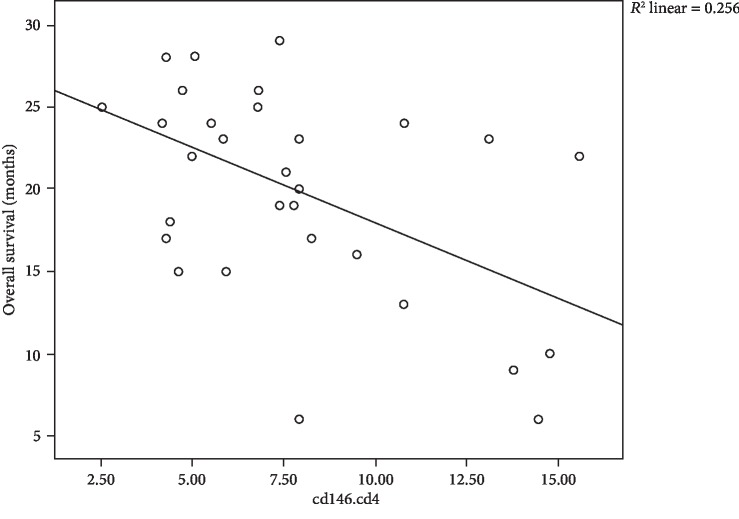
Scatter plot of the relation between CD146^+^CD4^+^ cells and OS of 31 children with B-cell ALL.

**Figure 4:**
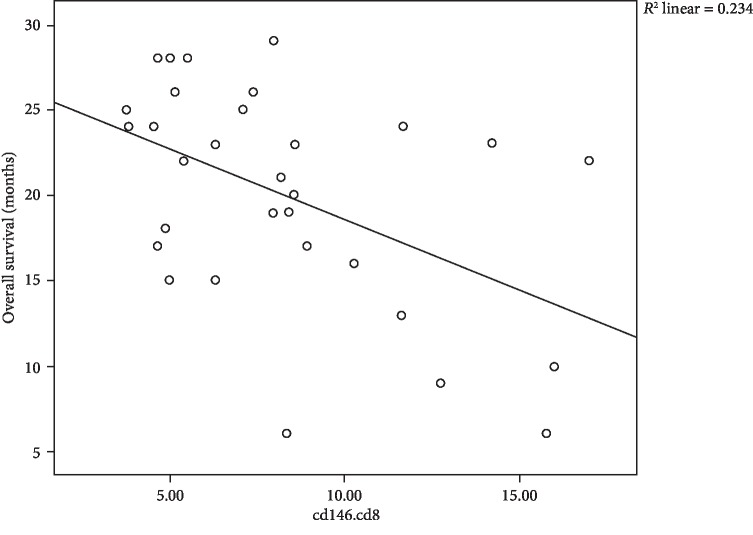
Scatter plot of the relation between CD146^+^CD8^+^ cells and OS of 31 children with B-cell ALL.

**Figure 5:**
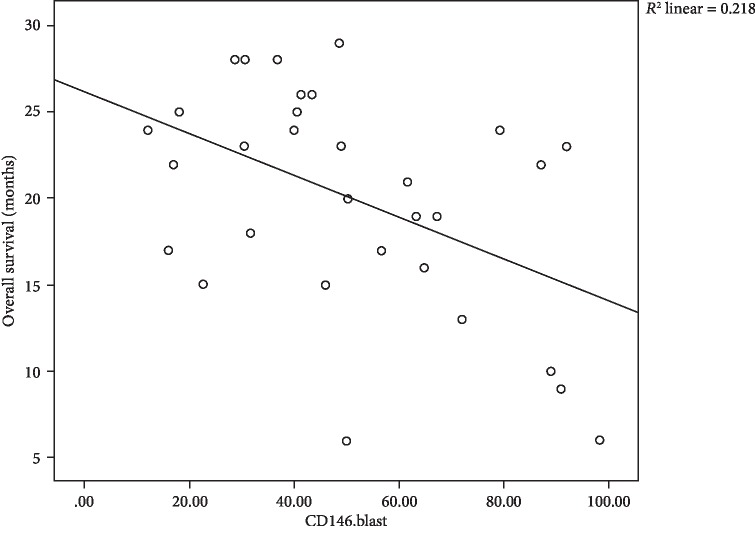
Scatter plot of the relation between CD146^+^ blasts and OS with 31 children with B-cell ALL.

In our results, we found negative correlations between CD4, CD8, and blasts expressing CD146 and overall survival, where moderate negative correlation between CD146^+^CD4^+^ cells and OS with *r* = −0.506 and *P* = 0.004 is shown Figure 3, moderate negative correlation between CD146^+^CD8^+^ cells and OS with *r* = −0.484 and *P* = 0.006 is shown in Figure 4, and in addition, moderate negative correlation between CD146^+^ blasts and OS with *r* = −0.466 and *P* = 0.008 is shown in Figure 5.

**Figure 6:**
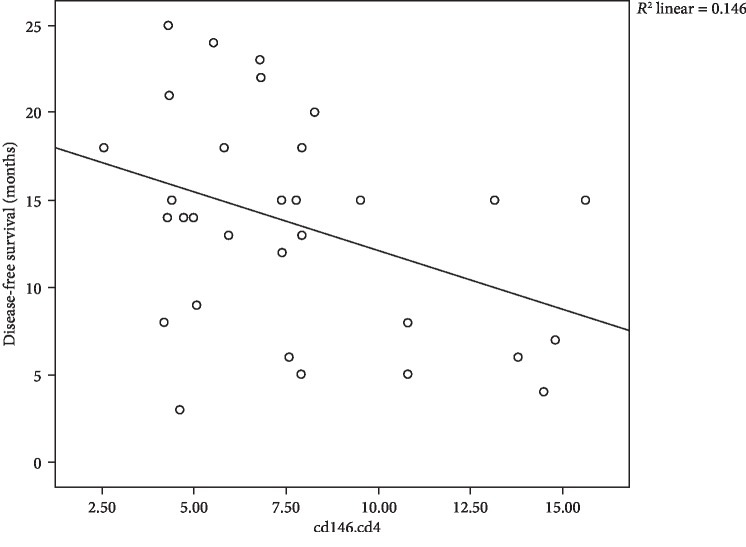
Scatter plot of the relation between CD146^+^CD4^+^ cells and DFS.

**Figure 7:**
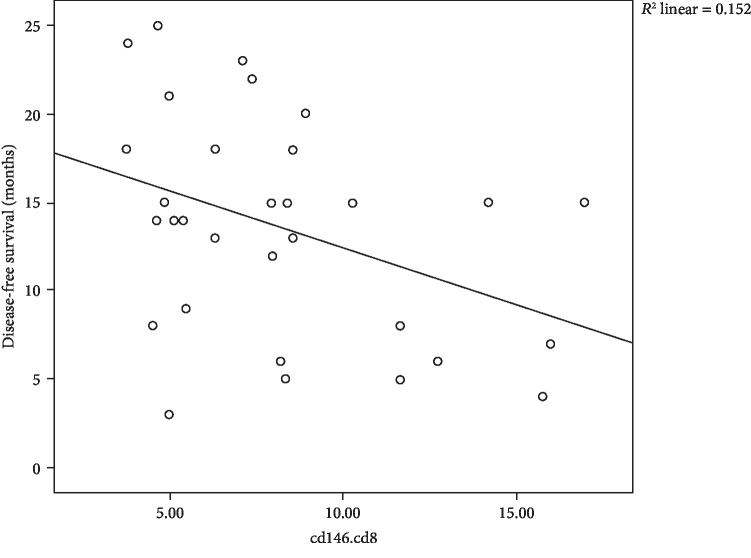
Scatter plot of the relation between CD146^+^CD8^+^ cells and DFS.

**Figure 8:**
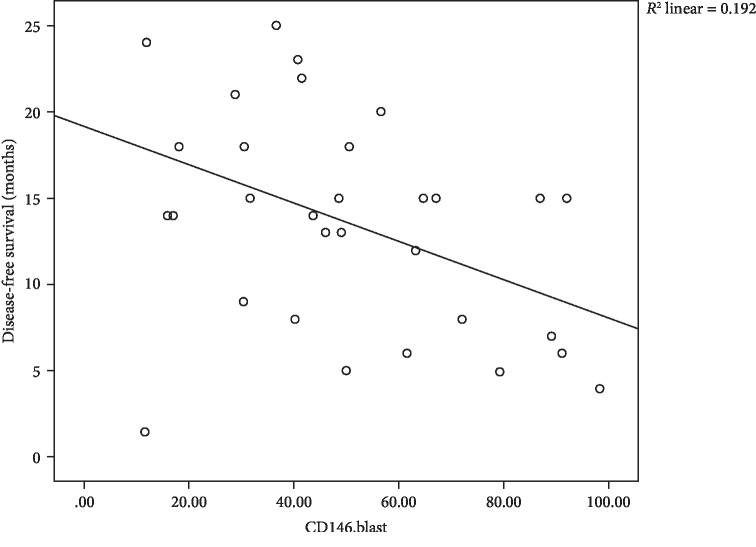
Scatter plot of the relation between CD146^+^ blasts and DFS.

With respect to disease-free survival (DFS), a significant negative impact of CD146^+^ cells on DFS with mild negative correlation between CD146^+^CD4^+^ cells and DFS with *r* = −0.382 and *P* = 0.034 is shown in Figure 6, also, negative mild correlation between CD146^+^CD8^+^ cells with the latter with *r* = −0.390 and *P* = 0.03 is shown in Figure 7, and lastly, moderate negative correlation between CD146^+^ blasts and DFS with *r* = −0.438 and *P* = 0.014 is shown Figure 8.

Intuitively, negative correlations between BM blasts and OS and DFS that lived up to significance (*P* < 0.012 for both) were evident; in addition, significantly negative correlations between CD34^+^ cells with OS and DFS (*P* < 0.001 and *P* < 0.033, respectively) are all demonstrated in Table 5.

**Figure 9:**
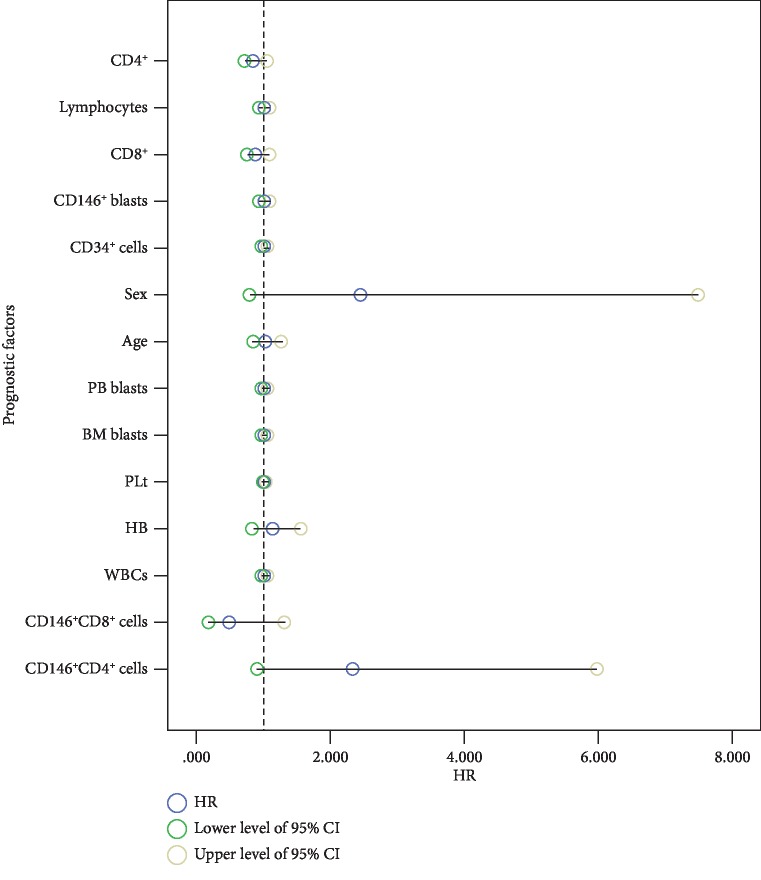
Forest plot of different prognostic factors of OS.

Upon doing Cox regression analysis, we found that only CD34^+^ cells were the most independent factor of OS (HR = 1.039, *P* = 0.014), while the HR of CD146^+^CD4^+^ cells was 2.326 (95%CI = 0.904‐5.982) and was considered the highest one among different immune cells followed by HR of CD146 blasts (HR = 1.023, 95%CI = 0.955‐1.096) (Table 6, Figure 9), as hazard ratio increased the risk of death from induction therapy increased.

### 3.3. DFS and OS according to Response to Induction Therapy

**Figure 10:**
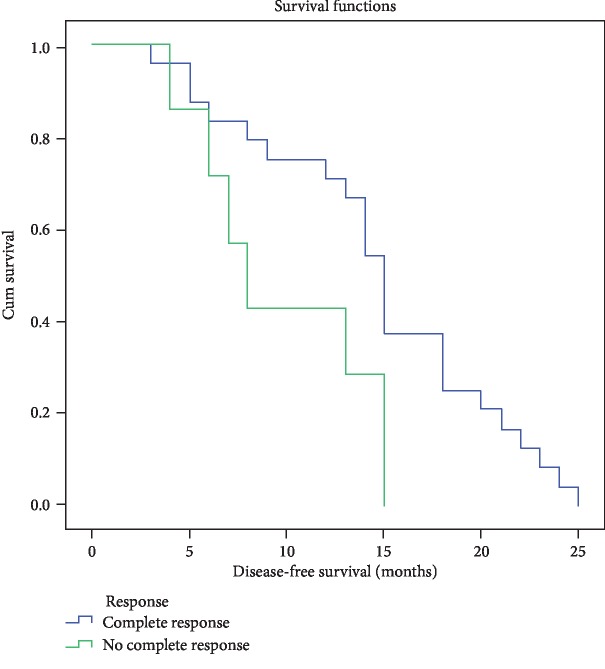
DFS for different response groups of B-cell ALL.

**Figure 11:**
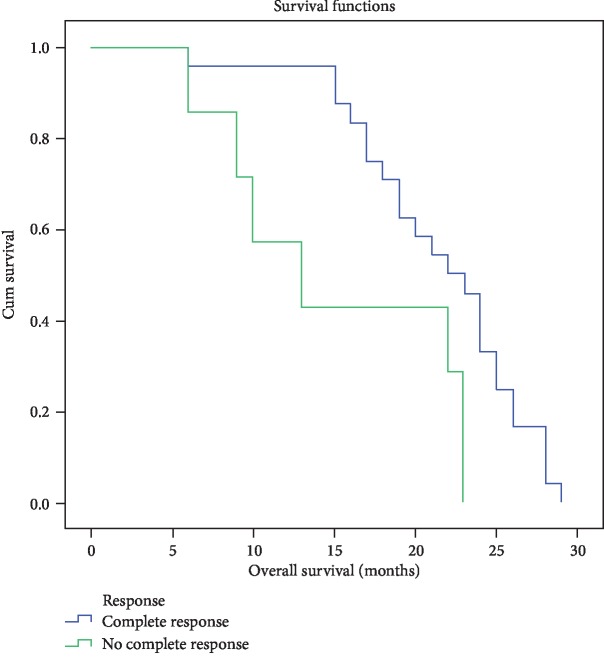
OS for different response groups of B-cell ALL.

Aside from the impact of immune cells expressing CD146 antigen, the median DFS for children responding completely to induction therapy was 15 ± 0.678 months (95%CI = 13.672‐16.328), while for those with less than complete response, the median DFS was 8 ± 1.309 (95%CI = 5.434‐10.566) with log‐rank = 4.513 and *P* = 0.034 (Figure 10). The median OS for the former group was 22 ± 1.633 (95%CI = 18.799‐25.201), while the median OS for the latter group was 13 ± 3.928 (95%CI = 5.301‐25.699), with log‐rank = 5.71, *P* = 0.017 (Figure 11).

## 4. Discussion

Advances in chemotherapy regimens, supportive care, and lastly immunotherapy of ALL resulted in a meaningful improvement of a 5-yr survival from 60% to approximately 90% for children more youthful than 15 years and from 28% to greater than 75% for adolescents elderly 15 to 19 years [22]. ALL is a disease of different prognostic factors including clinical, laboratory, genomic, cytogenetic, and immunophenotyping ones, and for efficient outcomes, adherence to risk-adapted therapy may be the way to reach the berth.

The notion to find more prognostic factors might decrease our losses of cases especially those expected to achieve better outcome; one of these factors is CD146 antigen that is expressed in various cell types to bind with unidentified ligand [5] exerting multifunctional activities as previously mentioned.

The results provided here were interesting and novel trying to uncover the role of CD146^+^ cells in this limited number of pediatric B-cell ALL, passing by significant accumulations of these cells in patients compared to controls to significant correlations between CD146^+^CD4^+^ cells, CD146^+^CD8^+^ cells, and CD146^+^ blasts with other poor prognostic features including CD34^+^, and BM blasts, to significant accumulations of these cells in children that did not gain CR, to finally unexpectedly worse impact of these cells on OS and DFS.

Contrary to Cavazzini et al. [20] who found that the expression of CD146^+^ cells was detected in 38.8% of B-cell ALL (14/38) of his retrospective series of 162 leukemic patients (ALL+AML), Xie et al. [21] tried to compare the expression rates of CD146 in adult and children's B-ALL patients which were 29.17% and 9.09%, respectively, showing that this difference was significant (*P* < 0.05). However, all our patients expressed CD146 with variable percentages, and the mean for CD146^+^CD4^+^ cells was 7.58 ± 3.517, the mean expression for CD146^+^CD8^+^ cells was 8.392 ± 3.798, and the mean expression for CD146^+^ blasts was 51.347 ± 24.133.

CD146 was one of the most significantly expressed genes having CD equivalent in B-cell ALL switched from B-lineage to monocytoid lineage (which represented 4-6% of B-cell ALL) [23].

In a previous study, CD146 expression strongly associated with Ph+ positivity in B-ALL with the highest percentage of CD146-positive blasts in all Ph-positive B-ALL cases [20]; in our study, we did not relate the percentage of CD146 expression with that of Ph chromosome possibly because of shortage of data collection and limited awareness of this relation.

Plenty studies in solid tumors showed that increased CD146 expression was associated with short survival outcomes in patients with various cancer types. Nevertheless, these studies were small numbered and less powered, so a meta-analysis of its role in different solid tumors was introduced that scientifically estimated survival data of 2,694 solid tumor adult patients included in 12 different studies. Overall, these results clearly indicated that high CD146 expression was a poor prognostic factor in solid tumors, including poor OS (pooled HR = 2.496, 95%CI = 2.115–2.946, *P* = 0.001) and poor TTP (pooled HR = 2.445, 95%CI = 1.975–3.027, *P* = 0.001) [17]. Upon stratifying the results by cancer type, the prognostic value of CD146 overexpression was significant in gastrointestinal and lung cancers.

Conversely, no study up-to-date evaluates adequately its role in hematologic malignancies; Xie et al. [21] explored the expression of CD146 in pediatric B-cell ALL compared to its counterpart in adults and found it to be related positively to CD64 and CD117 in adults, while in children's B-ALL, CD146 was positively related to CD71 and CD58 (*P* < 0.05). In addition, CD146 was significantly associated with lower response to induction therapy in both adult and pediatric B-ALL; we partially agreed with the previous study where CD146 expression was significantly associated with higher non-CR rate, but we did not correlate its expression with CD71 and CD58; instead, we correlated it with CD34, BM blasts, CD8^+^, CD4^+^, and lymphocytes.

In pediatrics, CD146 is widely expressed in embryonic tissues, including neural crest and its derivatives [24], but in hematopoietic tissue, it is expressed in a subpopulation of activated T cells corresponding to <1% of all cells in PB and BM. During development, CD146 is involved in various physiological functions, including cell migration, different cellular interactions, signaling, and morphogenesis.

In addition, Pujades et al. [24] demonstrated that CD146 expression is strongly associated with adverse clinical outcomes in malignancies derived from the neural crest linage including melanoma. More importantly, CD146^+^ cells were present in primary malignant rhabdoid tumors and exhibited exclusive *in vivo* tumorigenic potential sufficiently to suggest its utility as a potent biomarker for risk stratification and possibly treatment of malignant rhabdoid tumors.

Yet, the role of CD146 expression in pediatric B-ALL remains to be defined; we propose that CD146 will be considered for subsequent studies in pediatric ALL to better define leukemic subtypes and whether to be a potential target for subsequent therapy like melanoma.

Our study suffered from several limitations; small number of patients included in our study could be attributed to financial issues, the relation of CD146 expression to Philadelphia chromosome was missing, the difference of expression of CD146 among different risk groups of B-ALL was not touched, and by the same token, the study was not bolstered by CD146 relations to adverse genetic features including TCF3-PBX1 fusion, MLL gene rearrangement, t(17:19), hypodiploidy, and intrachromosomal amplification of chromosome 21.

## 5. Conclusion

CD146 was expressed significantly in children's B-ALL and associated with poor prognostic features including poor response and treatment outcomes and could be a possible poor prognostic factor in pediatric B-cell ALL.

## Figures and Tables

**Table 1:** Clinical data of ALL patients.

Data	Number (%)
Age (mean ± SD)	7.09 ± 2.88
Median	6.5 years
Range	3.3-12.6 years
Sex (male/female)	18/13 1.4 : 1
Fever (≥38.5°)	16 (51.6%)
LN enlargement	8 (25.8%)
Splenomegaly	7 (22.6%)
Hepatomegaly	10 (32.3%)

Data were expressed as number, percentage, and mean ± SD.

**Table 2:** Immune features of B-ALL patients and their controls with significance.

	Group	*N*	Mean	SD	SE	*P* value
cd146.cd4	Patients	31	7.5853	3.517	.632	0.001
	Controls	27	2.0168	.376	.072	

cd146.cd8	Patients	31	8.392	3.798	.682	0.005
	Controls	27	2.1782	.406	.0782	

WBC	Patients	31	32.43	23.618	4.242	NA
	Controls	NA	NA	NA	NA	

HG	Patients	31	7.7677	1.749	.31430	NA
	Controls	NA	NA	NA	NA	

PLT	Patients	31	100.94	103.66	18.617	NA
	Controls	NA	NA	NA	NA	

BM blast%	Patients	31	59.0323	21.97	3.94655	NA
	Controls	NA	NA	NA	NA	

PB blast%	Patients	31	20.0839	9.84	1.76618	NA
	Controls	NA	NA	NA	NA	

CD34	Patients	31	52.3613	21.523	3.865	NA
	Controls	NA	NA	NA	NA	

CD146.blast	Patients	31	51.3472	24.133	4.334	NA
	Controls	NA	NA	NA	NA	

CD8	Patients	31	10.3497	5.409	.9715	NA
	Controls	28	19.7607	10.509	1.986	

Lymphocytes	Patients	31	34.9600	8.88	1.594	0.005
	Controls	28	61.3578	12.21	2.306	

CD4	Patients	31	23.1857	4.856	.872	0.003
	Controls	28	39.8459	9.564	1.807	

NA: not applicable; BM: bone marrow; PB: peripheral blast. Data were expressed as number, mean, SD, SE, and independent sample *t*-test used for significance.

**Table 3:** Relations between CD146 and different flow cytometric immune cells and blasts of ALL.

		cd146.cd4	cd146.cd8	BM blast%	PB blast%	CD34	CD146.blast	CD8	CD4
cd146.cd4	*r*	NA	+0.999	+0.490	.017	+0.422	+0.910	.121	-.157
	Sig.		.001	.005	.926	.017	.001	.517	.398

cd146.cd8	*r*	0.999	NA	+0.494	.017	+0.420	+0.932	.121	-.157
	Sig.	.001		.001	.926	.018	.006	.517	.398

BM blast%	*r*	+0.490	+0.494	NA	-.072	+0.563	+0.528	-.197	-.082-
	Sig.	.005	.001		.701	.001	.002	.289	.660

PB blast%	*r*	.017	.017	-.072	NA	.092	-.043	-.001	-.145
	Sig.	.926	.926	.701		.624	.817	.995	.437

CD34	*r*	+0.422^∗^	+0.420	+0.56^∗^	.092	NA	+0.405^∗^	-.207	-.166
	Sig.	.017	.018	.001	.624		.024	.264	.372

CD146.blast	*r*	+0.919	+0.932	+0.528	-.043	+0.405^∗^	NA	.072	-.069
	Sig.	.007	.005	.002	.817	.024		.699	.712

CD8	*r*	.121	.121	-.197	-.001	-.207	.072	NA	-.474
	Sig.	.517	.517	.289	.995	.264	.699		.006

CD4	*r*	-.157	-.157-	-.082	-.145	-.166	-.069	-.481	NA
	Sig.	.398	.398	.660	.437	.372	.712	.007	

BM: bone marrow; PB: peripheral blast; NA: not applicable; *r*: Pearson correlation coefficient; Sig.; significance.

**Table 4:** Impact of the mean percentages of CD146^+^ cells in response to induction therapy.

CD146^+^ cell type	Response to induction therapy		*P* value < 0.05
	CR	<CR	
	Mean ± SD	Mean ± SD	
CD4^+^ cells	6.198 ± 1.96	12.929 ± 2.695	0.001
CD8^+^ cells	6.640 ± 2.12	13.684 ± 2.94	0.0004
Blasts	41.560 ± 18.06	82.626 ± 16.82	0.001

Data were expressed as mean ± SD and independent sample *t*-test for significance.

**Table 5:** Correlations between survival functions and different prognostic factors.

		BM blast%	PB blast%	CD34	CD8	Lymphocytes	CD4
OS	*r*	-.445	-.178	-.678	.248	.297	.207
	Sig.	.012	.337	.001	.178	.105	.263

DFS	*r*	-.443	-.103	-.383	.239	-.139	-.296
	Sig.	.012	.581	.033	.196	.457	.106

BM blast%	*r*	NA	-.072	.563	-.197	-.030	-.082
	Sig.		.701	.001	.289	.874	.660

PB blast%	*r*	-.072	NA	.092	-.001	-.060	-.145
	Sig.	.701		.624	.995	.749	.437

CD34	*r*	.563	.092	NA	-.207	-.259	-.166
	Sig.	.001	.624		.264	.159	.372

CD8	*r*	-.197	-.001	-.207	NA	.436	-.474
	Sig.	.289	.995	.264		.014	.007

Lymphocytes	*r*	-.030	-.060	-.259-	.436	NA	.252
	Sig.	.874	.749	.159	.014		.172

CD4	*r*	-.082	-.145	-.166	-.474	.252	NA
	Sig.	.660	.437	.372	.007	.172	

Data were analyzed using the Pearson correlation test. *r*: correlation coefficient; Sig.: significance.

**Table 6:** Cox regression analysis of different prognostic factors of OS.

	B	SE	Wald	df	Sig.	HR	95.0% CI for HR	
							Lower	Upper
cd146.cd4	.844	.482	3.067	1	.080	2.326	.904	5.982
cd146.cd8	-.745	.513	2.113	1	.146	.475	.174	1.296
WBC	.016	.018	.801	1	.371	1.016	.981	1.052
HG	.131	.155	.711	1	.399	1.140	.841	1.545
PLT	.003	.002	1.402	1	.236	1.003	.998	1.008
BM blasts	-.004	.019	.051	1	.821	.996	.959	1.034
PB blasts	.020	.029	.508	1	.476	1.021	.965	1.079
AGE	.027	.102	.070	1	.791	1.027	.841	1.256
SEX	.894	.572	2.440	1	.118	2.444	.796	7.503
CD34	.038	.015	6.100	1	.014	1.039	1.008	1.071
CD146.blast	.023	.035	.429	1	.512	1.023	.955	1.096
CD8	-.100	.086	1.369	1	.242	.905	.765	1.070
Lymphocytes	.009	.044	.040	1	.842	1.009	.925	1.101
CD4	-.139	.086	2.649	1	.104	.870	.735	1.029

Data were analyzed by the Cox regression test.

## Data Availability

All data of the study are available whenever requested.
